# ADAM‐1: A Novel AI driven approach to Alzheimer's disease Research and Multi‐modal Data Analytics

**DOI:** 10.1002/alz70855_100530

**Published:** 2025-12-23

**Authors:** Ziyuan Huang, Vishaldeep Kaur Sekhon, Ouyang Guo, Mark Newman, Roozbeh Sadeghian, Maria L Vaida, Cynthia Jo, Doyle Ward, Vanni Bucci, John P Haran

**Affiliations:** ^1^ UMass Chan Medical School, Worcester, MA, USA; ^2^ Johns Hopkins University, Baltimore, MD, USA; ^3^ Harrisburg University of Science and Technology, Harrisburg, PA, USA; ^4^ DevIS, Arlington, VA, USA; ^5^ University of Massachusetts Chan Medical School Chan Medical School, Worcester, MA, USA; ^6^ University of Massachusetts Chan Medical School, Worcester, MA, USA

## Abstract

**Background:**

Alzheimer's disease (AD) is a complicated neurodegenerative disorder influenced by dynamic interactions among clinical, microbial, and other complex underlying mechanisms. The Alzheimer's Disease Analysis Model Generation 1 (ADAM‐1) is an innovative multi‐agent large language model (LLM) framework proposed to deal with the complications of analyzing diverse and multi‐modal datasets. ADAM‐1 integrates clinical datasets, microbiome profiles, and existing Alzheimer's publications using retrieval‐augmented generation (RAG) techniques supporting AI agents to enhance diagnostic and analytical capabilities offering unified and comprehensive insights into Alzheimer's disease prognosis.

**Method:**

The study incorporates a multi‐modal dataset with paired clinical and gut microbiome data from 102 nursing home residents, including 64 healthy controls (HC) and 38 individuals with AD, collected across four facilities in central Massachusetts as part of one of our previous studies. ADAM‐1, built on the GPT‐4o‐mini‐2024‐07‐18 model, integrates three AI agents designed for Alzheimer's binary classification: a computational agent for generating descriptive statistics, a summarization agent for synthesizing insights from the data and knowledge database, and a classification agent for performing binary predictions based on prior outputs. The knowledge database comprises 80,909 Alzheimer's‐focused publications from PubMed. Classification performance was assessed using F1 scores across 15 randomized seeds, with comparisons to XGBoost as the baseline model. The study was conducted using Python 3.10.14 on an Ubuntu 24.04.1 LTS workstation with four 3090 GPUs.

**Result:**

For Alzheimer's classification, ADAM‐1 achieved a mean F1 score comparable to that of XGBoost (*p* = 0.0967, t‐test) while demonstrating significantly reduced F1 score variance (*p* = 0.0083, F‐test), indicating more stable performance across evaluations using 15 randomized seeds. The reduced variance in F1 scores emphasizes the reliability of ADAM‐1 in handling relatively small sample data, a common scenario in clinical translational research.

**Conclusion:**

ADAM‐1 offers a robust and consistent platform for multi‐modal data analysis in Alzheimer's research. The system's human‐machine interaction through natural language queries enhances data interpretability, expanding and broadening researchers' insights in analyzing such complex datasets. Future versions of ADAM will include blood biomarkers and neuroimaging thus enabling more comprehensive and precise diagnostics, advancing the understanding of the complicated and dynamic underlying mechanisms of Alzheimer's disease progression.

